# The effect of antenatal care follow-up on neonatal health outcomes: a systematic review and meta-analysis

**DOI:** 10.1186/s40985-018-0110-y

**Published:** 2018-12-17

**Authors:** Amsalu Taye Wondemagegn, Animut Alebel, Cheru Tesema, Worku Abie

**Affiliations:** 1 Department of Biomedical Sciences, School of Medicine, Debre Markos University, Debre Markos, Ethiopia; 2 Department of Nursing, College of Health Sciences, Debre Markos University, Debre Markos, Ethiopia; 3 Department of Public Health, College of Health Sciences, Debre Markos University, Debre Markos, Ethiopia

**Keywords:** Antenatal care, Maternal health service, Follow up, Visits, Utilizations, Neonatal mortality/death, Meta-analysis

## Abstract

**Background:**

Neonatal mortality is one of the major public health problems throughout the world and most notably in developing countries. There exist inconclusive findings on the effect of antenatal care visits on neonatal death worldwide. Thus, the aim of this systematic review and meta-analysis was to reveal the pooled effect of antenatal care visits on neonatal death.

**Methods:**

The present systematic review and meta-analysis was performed using published literature, which was accessed from national and international databases such as, Medline/PubMed, EMBASE, CINAHL, Cochrane Central library, Google Scholar, and HINARI. STATA/SE for windows version 13 software was used to calculate the pooled effect size with 95% confidence intervals (95% CI) of maternal antenatal care visits on neonatal death using the DerSimonian and Laird random effects meta-analysis (random effects model), and results were displayed using forest plot. Statistical heterogeneity was checked using the Cochran *Q* test (chi-squared statistic) and *I*^2^ test statistic and by visual examination of the forest plot.

**Results:**

A total of 18 studies, which fulfilled the inclusion criteria, were included in the present systematic review and meta-analysis. The finding of the present systematic review and meta-analysis revealed that antenatal care visits decrease the risk of neonatal mortality [pooled effect size 0.66 (95% CI, 0.54, 0.80)]. Cochrane *Q* test (*P* < 0.001) revealed no significant heterogeneity among included studies, but *I*^2^ statistic revealed sizeable heterogeneity up to 80.5% (*I*^2^ = 80.5%). In the present meta-analysis traditional funnel plot, Egger’s weighted regression (*P* = 0.48) as well as Begg’s rank correlation statistic (*P* = 0.47) revealed no evidence of publication bias.

**Conclusions:**

The present systematic review and meta-analysis revealed that antenatal care visits were significantly associated with lower rates of neonatal death. The risk of neonatal death was significantly reduced by 34% among newborns delivered from mothers who had antenatal care visits. Thus, visiting antenatal care clinics during pregnancy is strongly recommended especially in resource-limited settings like countries of sub-Saharan Africa.

**Electronic supplementary material:**

The online version of this article (10.1186/s40985-018-0110-y) contains supplementary material, which is available to authorized users.

## Background

Neonatal mortality is defined as the death of a newborn in the first 4 weeks of life (neonatal period), and it is expressed in terms of rate of neonatal deaths per 1000 live births [1]. Neonatal mortality is one of the major public health problems throughout the world, most notably in developing countries. Globally, an estimated number of 2.6 million neonatal deaths occurred in 2016, accounting for 46% of deaths among under-five children [2, 3]. Almost all (99%) newborn deaths occur in low- and middle-income countries. Moreover, Africa and South Asia have made the least progress in reducing neonatal deaths [1]. Sub-Saharan Africa (SSA) carries the highest neonatal mortality in the world and achieved the lowest progress in the reduction of neonatal mortality [4].

One of the targets of the Sustainable Development Goals (SDGs) fixed by the United Nations in 2015 is to end preventable deaths of newborns, with all countries aiming to reduce neonatal mortality to as low as 12 deaths per 1000 live births by the year 2030 [2]. Globally, neonatal mortality declined from 5.1 million in 1990 to 2.6 million in 2016, but this decline in neonatal mortality over 1990–2016 has been slower than that of post-neonatal under-five mortality (1–59 months): 47%, compared with 58% globally. This pattern applies to most low- and middle-income countries [2]. Evidence suggests that about 75% of neonatal deaths in developing countries could be prevented with simple and low-cost tools that already exist, such as antibiotics for pneumonia and sepsis, sterile blades to cut umbilical cords, and using knit caps and kangaroo care to keep babies warm [5, 6]. These modifiable risk factors could be avoided through implementation of preventive measures like antenatal care (ANC) services [7].

ANC is one of the fundamental strategies recommended to reduce the risk of neonatal mortality in any community, despite socio-demographic background [8–11]. ANC improves the survival and health of babies directly by reducing stillbirths and neonatal deaths and indirectly by providing an entry point for health contacts with the mother at a key point in the continuum of care [12]. In addition, it will help the health professionals to identify women at increased risk of adverse pregnancy outcomes. The World Health Organization (WHO) recommends at least eight ANC visits to provide effective ANC services, particularly in low-income countries [13].

In different parts of the world, several studies have been conducted to determine the effect of ANC on neonatal mortality. The findings reported from these studies were controversial and inconclusive in nature. In most studies, providing ANC services reduced the risk of neonatal mortality. In others, the ANC visits were not significantly associated with neonatal mortality. For better intervention, current and up-to-date information regarding the effect of ANC on neonatal survival is crucial, especially in low- and middle-income countries where most neonatal deaths occur. However, in recent years, despite small studies, there has been no worldwide study to determine the effect of ANC on neonatal mortality. Therefore, this systematic review and meta-analysis aims to estimate the effect of ANC follow-up on neonatal mortality using available studies. The findings from this systematic review will highlight the effect of ANC interventions, with implications to improve health workers’ operations and cost-effectiveness and to accelerate the reduction of neonatal deaths.

## Methods

### Searching strategies

The present systematic review and meta-analysis was conducted to quantify the pooled effect of ANC on neonatal mortality. Literature was reviewed from national and international databases. The following databases were systematically searched: Medline/PubMed, EMBASE, CINAHL, Cochrane Central library, Google Scholar, and HINARI (Health Inter Network Access to Research Initiative) from August 29 to October 30, 2017. The reports were accessed using the following key terms/like Mesh terms, Emtree/: “antenatal care/ANC,” “maternal health service,” “follow up,” “visits,” “utilizations,” “neonatal death,” and “newborn care.” The key terms were used individually and in combination through “AND” and “OR.” In addition, after identification of studies and review articles, their lists of reference were searched to identify more eligible studies. The above database search strategy and terms are presented in (Additional file 1: Table S1). This systematic review and meta-analysis used the PICO (Population, Intervention, Comparison and Outcomes) framework to determine the eligibility of the articles included. The study Population (P) were neonates (age < 28 days), the Intervention (I) was focused ANC follow-up, the Comparison (C) group were neonates born from mothers who did not have ANC follow-up, and the Outcomes (O) of this study were the occurrence of death within 28 days after delivery.

### Study selection

Potentially eligible studies for this systematic review and meta-analysis were selected in three stages: titles alone, abstracts, and then full-text articles, based on inclusion criteria: All quantitative studies reported in English language, published in peer-reviewed journals, and revealed the association between antenatal care visits and neonatal deaths were included.

However, studies which did not report the maternal ANC visit status as well as outcomes of their newborns were excluded. In addition, articles, which were not fully accessible, after at least two-email contact with the primary authors, were excluded. Exclusion of these articles is because of the inability to assess the quality of articles in the absence of full text.

Moreover, review articles, commentary, and editorials were excluded from analysis, but read to identify eligible articles for the current review. Potentially eligible articles were identified by two reviewers (ATW and AA), through independent reading of the titles and abstracts, which were searched and accessed broadly. The full texts of these articles were accessed, and independent assessment was carried out by two reviewers, ATW and AA, for eligibility based on the predetermined inclusion and exclusion criteria. Discrepancies between the reviewers were resolved through discussion and common consensus of all investigators. Multiple publications of the same study were not identified.

### Data abstraction and quality assessment of the studies

Two reviewers (ATW and AA) independently extracted data, which was then confirmed by the other investigators (CT and WA). The data extraction was performed using the following template: first author, year of publication, study setting, study design, sample size, number of survivors and died neonates among ANC visitor mothers, and number of survivors and died neonates among those mothers without ANC visits. Controversies during the data extraction process were resolved through discussion and common understanding among the reviewers. To the researchers’ knowledge, there exist no well-defined tools for assessing quality of observational epidemiological studies [14]. However, sample size, sampling method, and response rate were considered to assess the quality of included studies. The studies reported using larger sample size (reported outcomes on at least 50 participants), random sampling, and higher response rate (studies with response rate greater than 80%) were considered as high-quality studies.

### Statistical analysis

Data were extracted from each eligible article using a template prepared in Microsoft Excel spreadsheet software and imported into STATA/SE for windows Version 13 software for further analysis. Stata version 13 was used to calculate the pooled effect size with 95% confidence intervals (95% CI) of maternal ANC visit status on neonatal death using the DerSimonian and Laird [15] random effects meta-analysis (random effects model).

### Assessing statistical inconsistency and publication bias

Statistical heterogeneity was weighed using the Cochran *Q* test (chi-squared statistic) and *I*^2^ test statistic and by visual examination of the forest plot (overlap of confidence intervals). Cochran’s *Q* test was used to test the null hypothesis of no significant heterogeneity across the studies [16]. Cochran’s *Q* is calculated as the weighted sum of squared differences between individual study effects and the pooled effect across studies, with the weights being those used in the pooling method. Cochran’s *Q* statistic follows a chi-squared distribution with *k* − 1 degree of freedom where *k* is the number of studies. Cochran’s *Q* statistical heterogeneity test is considered as statistically significant at *P* < 0.10. The *I*^2^ statistic, the percentage of variation (inconsistency) in the measures of association across studies that is due to heterogeneity rather than chance, [17] was also estimated. The *I*^2^ statistic is equal to the quantity of Cochran’s *Q* minus its degree of freedom (df) divided by Cochran’s *Q* times 100%, or *I*^2^ = 100% × (*Q* − df)/*Q*. The value of *I*^2^ ranges between 0 and 100%, where 0% indicates no observed heterogeneity and large values indicate increasing heterogeneity [17]. An *I*^2^ value of 25%, 50%, and 75% is considered as low, moderate, and high heterogeneity [17]. Each study’s risk ratio (RR) with 95% confidence intervals (CIs) was presented on traditional funnel plots and Begg’s funnel plot to reveal publication bias. In addition, Egger’s weighted regression and Begg’s rank correlation tests were used to check for publication bias (*P* < 0.05 is considered statistically significant). Cumulative meta-analysis was also run to assess the effect of each study on the pooled estimate.

## Results

### Accessed studies

**Fig. 1:**
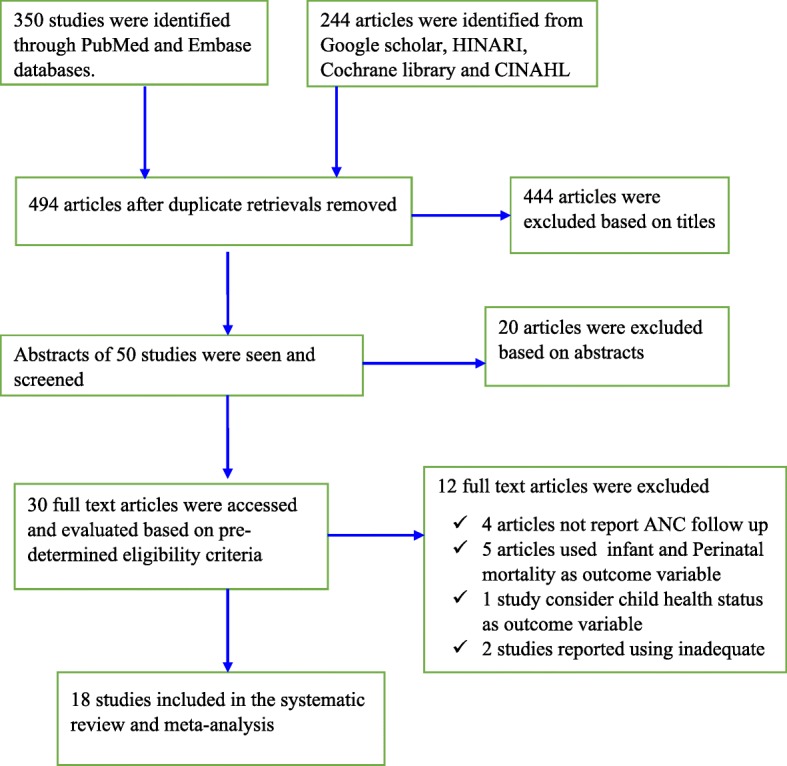
Flow chart, which reveals the procedures of study selection for the current systematic review and meta-analysis

The reports of the present meta-analysis was presented based on the Preferred Reporting Items for Systematic Reviews and Meta-Analyses (PRISMA) guideline [18] (Additional file 2: Table S2). A total of 594 articles related to the review titles were accessed in our initial literature search. We removed duplicate retrievals, and 494 articles remained. Upon initial screening, 444 articles were excluded by their titles which were found to be non-pertinent because of one of the following reasons: the titles of most of the papers were not directly related to the present topic, the titles of some of the papers consider an individual predictor other than ANC follow-up as exposure variable for neonatal health, and the titles of the remaining paper were for reviews in other topic areas. Of the remaining 50 articles, abstracts were screened and 20 articles were excluded because maternal health service utilization status was not considered as one of the exposure variables (predictor) on child health. For the remaining 30 papers, full-text articles were accessed and evaluated for eligibility based on predetermined inclusion and exclusion criteria and 12 studies were excluded. This was due to some of the articles only reporting the effect of delivery status and postnatal care on neonatal health status and other reports using infant and perinatal mortality as an outcome variable. Finally, 18 studies that fulfilled the inclusion criteria were included in the present systematic review and meta-analysis (Fig. 1).

### Description of background characteristics and outcome variables of included studies

Regarding study design, half (9) of the studies included in this systematic review and meta-analysis were cross-sectional studies [19–27], four were case-control studies [28–31], four cohort studies [32–35], and one community trial study [36]. The publication year of the included studies ranged between 2009 and 2017. The sample size of individual included studies ranged from 198 in a case-control study in Nepal to 56,307 in a cross-sectional study in Nigeria. Among the 18 included studies, almost all (94%) were from African and Asian low- and middle-income countries. Twelve studies were from SSA: Ethiopia [19, 31–33, 35], Kenya [26, 28], Malawi [22], Burkina Faso [36], Cameroon [34], Uganda [23], and Nigeria [24]. Five studies were from Asia: India [21], Iraq [27], Nepal [20, 29], and Pakistan [25]. Only one study was obtained from South America, Brazil [30]. In the current meta-analysis, a total of 94,118 live births were involved. From all live births, 3097 died within 28 days of birth, with a neonatal mortality rate of 32.91 per 1000 live births, and the remaining 91,021 survived beyond the first 28 days after birth. The total number of live births born from mothers who did not attend ANC visits was 49,706, and the total number of live births born to mothers who attended ANC follow-up was 44,412. Further descriptions of the characteristics and outcomes of this systematic review and meta-analysis are presented in Table 1.

**Table 1:** Summary of 18 studies included in the meta-analysis to determine the effect of ANC visits on neonatal mortality

Primary author (publication year)	Study setting/country	Study design	Sample size	No ANC visits		ANC visits	
				Survivors	Neonatal death	Survivors	Neonatal death
Kumar et al. (2014) [21]	India	Cross sectional	14,293	10,978	224	1824	37
Al-Ani et al. (2009) [27]	Iraq	Cross sectional	3249	1925	57	1242	25
Shah et al. (2015) [29]	Nepal	Case-control	198	10	17	69	57
Koffi et al. (2015) [22]	Malawi	Cross sectional	24,000	13,971	189	9709	131
Dahiru T (2015) [24]	Nigeria	Cross sectional	56,307	9196	222	10,577	215
Worku et al. (2014) [33]	Ethiopia	Cohort	763	452	10	255	10
Arunda et al. (2017) [26]	Kenya	Cross sectional	14,190	539	24	8074	87
Nascimento et al. (2012) [30]	Brazil	Case-control	396	70	38	194	94
Paudel et al. (2013) [20]	Nepal	Cross sectional	12,674	1988	49	2036	27
Ayaz et al. (2010) [25]	Pakistan	Cross sectional	565	253	4	297	11
Diallo et al. (2011) [36]	Burkina Faso	Community trial	1162	226	12	598	28
Ndombo et al. (2017) [34]	Cameroon	Cohort	332	86	30	194	22
Yego et al. (2017) [28]	Kenya	Case-control	600	227	105	133	13
Kananura et al. (2016) [23]	Uganda	Cross sectional	2237	1112	57	866	18
Worku et al. (2012) [32]	Ethiopia	Cohort	3789	205	106	2556	732
Wakgari et al. (2013) [19]	Ethiopia	Cross sectional	17,817	5506.4	246.9	2846.07	48.93
Kolola et al. (2016) [31]	Ethiopia	Case-control	336	119	46	122	26
Debelew et al. (2014) [35]	Ethiopia	Cohort	3604	1362	44	1204	34

### Findings of heterogeneity and publication bias of included studies

**Fig. 2:**
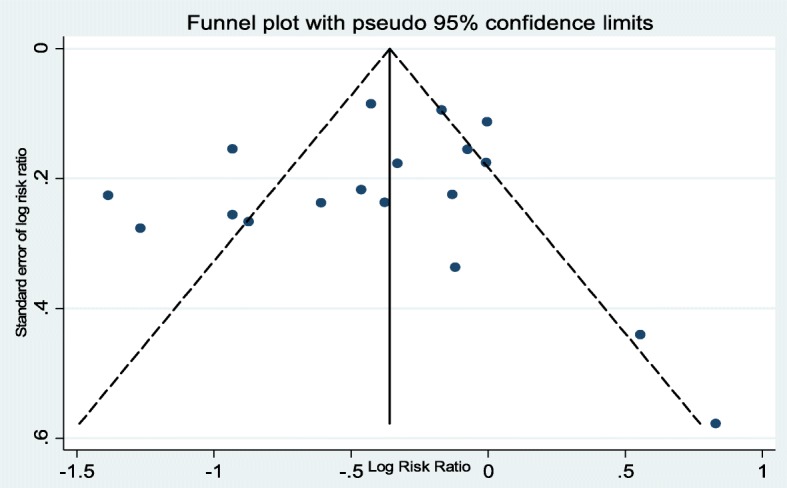
Traditional funnel plot of 18 included studies of the effect of ANC on neonatal death; the horizontal line refers the effect estimate, and the vertical line refers the expected 95% confidence intervals

**Fig. 3:**
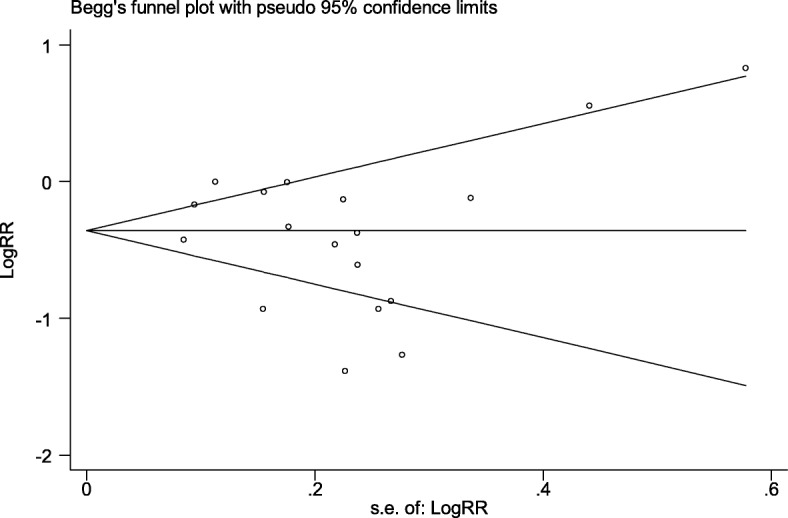
Begg’s funnel plot of 18 included studies of the effect of ANC visits on neonatal death; the horizontal line in the plot refers to the natural logarithm of effect estimate, and the vertical line refers the expected 95% confidence intervals

Analysis of included studies revealed heterogeneity using the Cochrane *Q* test statistic [*χ*^2^ = 87.2 (df = 17) *P* < 0.001]. In addition, sizeable heterogeneity was found to be up to 80.5% using the *I*^2^ test statistic (*I*^2^ = 80.5%). Because of this, a random effects meta-analysis model was used to quantify the effect of ANC follow-up on neonatal mortality. The effect estimates were distributed symmetrically on a traditional funnel plot (Fig. 2), indicating that there was no evidence of publication bias. Moreover, to ascertain this Begg’s funnel plot (Fig. 3), Begg’s rank correlation test was conducted, and the result of the test statistics revealed that there was no statistically significant bias with Kendall’s score of − 19 and *P* = 0.47. More importantly, Egger’s weighted regression test statistic was considered, revealing that there was no significant evidence of publication bias with *r* = − 0.88 (95% CI = − 3.47, 1.72) and *P* = 0.48.

### The effect of ANC follow-up on neonatal mortality

**Fig. 4:**
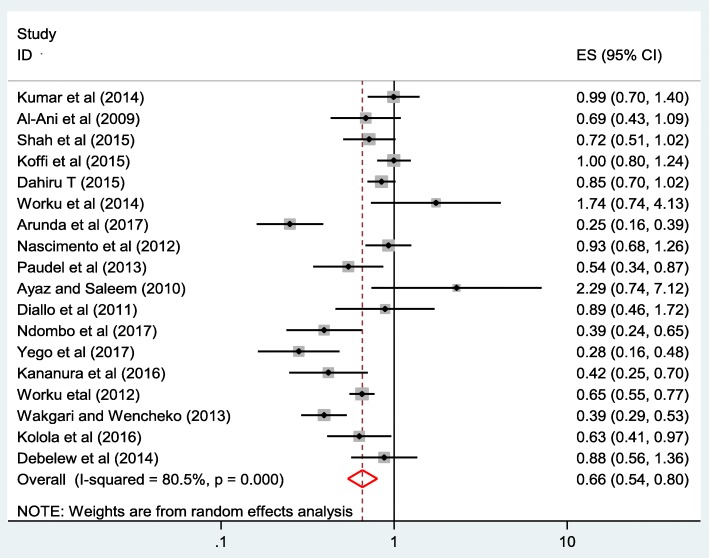
Forest plot of 18 included studies, which reveal the effect of ANC visits on neonatal death. The size of the square is proportional to the precision of the study-specific effect estimates, and the bars indicate the corresponding 95% CIs. The diamond is centered on the summary ES of all included studies, and the width indicates the corresponding 95% CI

The pooled effect size of neonatal death among those live births born to mothers who had ANC visits was 0.66 (0.54, 0.80) compared to those born to mothers without having ANC visits in the random effects model. The finding of the present systematic review and meta-analysis revealed that ANC visits decrease the risk of neonatal mortality by 34% (Fig. 4).

### Subgroup analysis

To decrease sizeable heterogeneity, subgroup analysis was performed based on the study design, sample size, and study settings. Accordingly, in the random effects model, cross-sectional and case-control studies were found to reveal significant effect size and the remaining cohort and community trial studies did not reveal significant effect size. The present meta-analysis also revealed different effect size with different sample size, and the higher the sample size the more precise the effect size. Finally, the setting in which the included studies were conducted was an important variable that contributed to the effect size differences in the random effects model. Studies conducted in SSA revealed statistically significant effect size (Table 2).

**Table 2:** Subgroup analysis of 18 included studies in this systematic review and meta-analysis considering study design, sample size, and study settings

Variables used for subgroup analysis		Random effects RR with (95%CI)
Study design	Cross sectional	0.64 (0.46, 0.88)
	Case-control	0.61 (0.40, 0.94)
	Cohort	0.72 (0.48, 1.07)
	Community trial	0.89 (0.46, 1.72)
Sample size	< 1000	0.7 (0.47, 1.05)
	1000–10,000	0.67 (0.55, 0.82)
	> 10,000	0.61 (0.41, 0.9)
Study settings	SSA	0.59 (0.46, 0.76)
	Asia	0.78 (0.58, 1.06)
	South America	0.93 (0.68, 1.25)

## Discussion

Globally, neonatal mortality remains a major concern, despite numerous interventions that have been made to improve the survival of newborns in recent years. The current systematic review and meta-analysis is perhaps the first of its kind to be conducted at the global level to examine the effect of ANC on neonatal mortality. The findings of this study will have important implications for maternal and child health programs run by governmental and non-governmental organizations. The findings of this meta-analysis revealed that ANC follow-up has a significant effect on neonatal mortality. In this meta-analysis, as most of the included studies were obtained from low- and middle-income countries, the result could be better applied for these countries. The meta-analysis indicated that neonatal mortality could be reduced by 34% through the implementation of ANC follow-up. This finding is consistent with a Demographic and Health Survey (DHS)-based study conducted in SSA countries to assess antenatal care and newborn survival [37].

It is well-known that ANC visits may help to reinforce maternal education and compliance, and provide an opportunity for screening for warning signs of pregnancy complications and treatment of infections [38]. In addition, ANC provides an important opportunity for health workers to teach mothers how to recognize warning signs of complications during pregnancy, labor, and delivery and encourage them to plan clean and safe deliveries preferably with trained assistance [39, 40]. Furthermore, during ANC follow-up, health care providers can provide information on postpartum care, newborn care, breastfeeding, problem signs, and appropriate action to be taken [41].

In this meta-analysis, to identify the possible sources of heterogeneity, we performed subgroup analysis based on the regions, sample size, and study design. The result of this subgroup analysis noted that the effect of ANC is statistically significant in SSA countries where more than 99% of worldwide neonatal deaths occurred [1]. The result obtained from this subgroup analysis indicated that 41% of neonatal mortality in SSA could be prevented through the implementation of ANC. The possible explanation for this variation in the effect of ANC on neonatal mortality might be due to the difference in ANC coverage across the world. In SSA, there is relatively low ANC coverage as compared to other regions of the world [42]. In areas where there is low coverage of ANC, mothers usually did not have access to ANC services. Therefore, pregnant women may not receive adequate health education regarding warning signs of pregnancy complications and often only go to a health facility after encountering difficulties during labor. As a result, infants are more likely to have health problems and die during the neonatal period.

### Limitations

Only English articles or reports were considered to conduct this review. In addition, the nature of the design of the included studies and the adequacy sample size might affect the estimated report. Furthermore, in this meta-analysis, the majority of the included studies were reported from developing countries, especially SSA and Asia. Therefore, the result may only be representative of the above regions.

## Conclusions and recommendations

The present systematic review and meta-analysis revealed that ANC visits were significantly associated with lower rates of neonatal death. The risk of neonatal death was significantly reduced overall by 34% among those newborns of mothers who attended ANC visits and more so (41%) in SSA. Thus, visiting ANC clinics during pregnancy is strongly recommended especially in resource-limited settings such as countries of SSA.

## Additional files

Additional file 1: **Table S1.** Search strategy for the MEDLINE/PubMed, Embase, Google Scholar and other databases used to access those articles which reveal effect of ANC follow-up on neonatal health outcomes. (DOCX 12 kb)

Additional file 2: **Table S2.** PRISMA checklist. (DOC 65 kb)

## Abbreviations

ANC: Antenatal care
CI: Confidence interval
ES: Effect size
NM: Neonatal mortality
RR: Risk ratio
SSA: Sub-Saharan Africa
