# Supplementary material for: The effect of antenatal care follow-up on neonatal health outcomes: a systematic review and meta-analysis
Source: Public Health Rev. 2018 Dec 17;39:33. doi: 10.1186/s40985-018-0110-y (PMC6296103; doi:10.1186/s40985-018-0110-y)
Supplement: Supplementary file 1 — Table S1. Search strategy for the MEDLINE/PubMed, Embase, Google Scholar and other databases used to access those articles which reveal effect of ANC follow-up on neonatal health outcomes. (DOCX 12 kb) [file 40985_2018_110_MOESM1_ESM.docx]

S1 Table. Search strategy for the MEDLINE/PubMed, Embase, Google Scholar and other databases used to access those articles which reveal effect of ANC follow-up on neonatal health outcomes

| Databases | Searching terms | Number of studies |
| --- | --- | --- |
| Google scholar | “effect” and “ANC” or “Prenatal care” and “follow-up” or “supplements” and “neonatal” or “newborn” and “outcomes” or “deaths” or “mortality” | 150 |
| MEDLINE/ PubMed and Embase | effect[All Fields] AND ("prenatal care"[MeSH Terms] OR ("prenatal"[All Fields] AND "care"[All Fields]) OR "prenatal care"[All Fields] OR ("antenatal"[All Fields] AND "care"[All Fields]) OR "antenatal care"[All Fields]) AND follow[All Fields] AND up[All Fields] AND ("infant health"[MeSH Terms] OR ("infant"[All Fields] AND "health"[All Fields]) OR "infant health"[All Fields] OR ("neonatal"[All Fields] AND "health"[All Fields]) OR "neonatal health"[All Fields]) AND outcomes[All Fields] | 350 |
| From other databases |  | 94 |
| Total retrieved articles |  | 594 |
| Final included studies |  | 18 |
